# Tunable Collagen I Hydrogels for Engineered Physiological Tissue Micro-Environments

**DOI:** 10.1371/journal.pone.0122500

**Published:** 2015-03-30

**Authors:** Elizabeth E. Antoine, Pavlos P. Vlachos, Marissa N. Rylander

**Affiliations:** 1 Department of Mechanical Engineering, Virginia Tech, Blacksburg, Virginia, United States of America; 2 School of Mechanical Engineering, Purdue University, West Lafayette, Indiana, United States of America; 3 Department of Mechanical Engineering, The University of Texas at Austin, Austin, Texas, United States of America; National University of Ireland, Galway (NUI Galway), IRELAND

## Abstract

Collagen I hydrogels are commonly used to mimic the extracellular matrix (ECM) for tissue engineering applications. However, the ability to design collagen I hydrogels similar to the properties of physiological tissues has been elusive. This is primarily due to the lack of quantitative correlations between multiple fabrication parameters and resulting material properties. This study aims to enable informed design and fabrication of collagen hydrogels in order to reliably and reproducibly mimic a variety of soft tissues. We developed empirical predictive models relating fabrication parameters with material and transport properties. These models were obtained through extensive experimental characterization of these properties, which include compression modulus, pore and fiber diameter, and diffusivity. Fabrication parameters were varied within biologically relevant ranges and included collagen concentration, polymerization pH, and polymerization temperature. The data obtained from this study elucidates previously unknown fabrication-property relationships, while the resulting equations facilitate informed *a priori* design of collagen hydrogels with prescribed properties. By enabling hydrogel fabrication by design, this study has the potential to greatly enhance the utility and relevance of collagen hydrogels in order to develop physiological tissue microenvironments for a wide range of tissue engineering applications.

## Introduction

Hydrogels using collagen I obtained from native tissues are in widespread use as scaffolds for 3D cell culture and tissue engineering. Historically, while desirable for tissue engineering due to their natural biocompatibility and similarity to native extracellular matrix (ECM) [1],collagen hydrogels have been at a disadvantage when compared with synthetic ECM mimics because of their complex nature [2]. In order to effectively use collagen hydrogels for tissue engineering, the ability to optimize hydrogel material properties to better replicate those of the target tissue and therefore provide physiological cues for regulation of cell behavior is essential [3]. However, this is challenging because the properties of collagen hydrogels depend on a broad range of fabrication parameters, including but not limited to source tissue, solubilization method, pH and temperature of polymerization, solution components, ionic strength, and collagen concentration [4].

Significant efforts have been devoted to characterization of functionally relevant bulk properties of collagen hydrogels to enable hydrogel design for specific applications. ECM properties including compression modulus, fiber structure, and diffusivity of bioactive molecules have been investigated [5–10]. Compression modulus is one of the key bulk properties used for tuning engineered scaffolds, as it regulates cell response, e.g. adhesion, differentiation, morphology, and migration [11–13]. The elastic moduli of hydrated biological tissues vary from 10^2^ to 10^6^ Pa [14]; therefore, collagen hydrogels spanning a similar range are desirable. Fiber structure, another important factor for designing tissue-mimicking hydrogels, regulates cell morphology and migration [8,12,15]. In collagenous *in vivo* tissues, pore size is generally heterogeneous and can vary from 1 to 20 μm [8]. Nutrient transport and drug delivery within tissues are generally limited by diffusivity, which depends on properties of the tissue and the diffusing molecule.

Although some correlations between hydrogel material properties and fabrication parameters have been demonstrated, the wide variation in fabrication protocols and characterization techniques used by different groups result in data which is difficult to interpret and often contradictory [4]. Furthermore, the relative influence of fabrication parameters on hydrogel properties cannot be easily determined from existing data, making it difficult to select an optimal set of fabrication parameters for a given application.

This study aims to fill this gap and measure the response of a set of hydrogel material properties to several important fabrication parameters, varied within ranges that correspond to biological applications. The fabrication parameters under consideration here were (1) collagen concentration, (2) polymerization temperature, (3) polymerization pH, and (4) molecule size (for diffusion measurements only). Collagen concentrations of 4, 6, 8, and 10 mg/ml (0.4, 0.6, 0.8, and 1.0% wt.) were studied because this range has been shown to support microfabrication and cell culture while matching concentrations which are found in natural tissues [5,16,17]. Temperatures of 23°C and 37°C were selected because they are easily attainable in tissue engineering laboratories and support cell viability during polymerization. A pH range of 7.4–8.4 was selected because this range supports cell viability [18,19] and several previous studies have suggested that pH can be a valuable tool for controlling hydrogel material properties [7,10,12]. Finally, fluorescently labeled dextrans with hydrodynamic radii mimicking cytokines and other bioactive molecules (1.4, 4.5, 6.0, and 8.5 nm) were used for diffusion studies. The parameter space is outlined in Table 1.

**Table 1:** Fabrication parameters varied in experiments.

Parameter	Values	Units
Concentration *C*	4, 6, 8, 10	mg/ml
Temperature *T*	23, 37	C
*pH*	7.4, 7.9, 8.4	-
Hydrodynamic Radius *r*_*H*_ *(*Molecular Weight *MW)*	1.4 (4), 4.5 (40),6.0 (70), 8.5 (150)	nm (kDa)

The properties characterized were polymerization kinetics, compression modulus, fiber structure, and diffusivity. These data formed the basis for empirical models of fabrication/property relationships for collagen I hydrogels as well as a sensitivity analysis which identifies the relative influence of each fabrication parameter on material properties.

## Experimental Methods

Collagen solutions were prepared using acid-soluble collagen I extracted from rat tail tendon at concentrations of 8, 12, 16, and 20 mg/ml and pH of 7.4, 7.9, and 8.4. Collagen solutions were mixed and pipetted into a variety of forms (cuvettes for spectrophotometry, confined chambers for compression measurement, and multi-well slides for fiber structure and diffusivity measurements) and subsequently polymerized at either 23°C or 37°C. For diffusivity measurements, FITC-labeled dextrans of 4, 40, 70, and 150 kDa were added to the collagen solutions before polymerization. A detailed description of collagen extraction and hydrogel fabrication can be found in S1 Methods.

### Polymerization Kinetics

**Fig 1:**
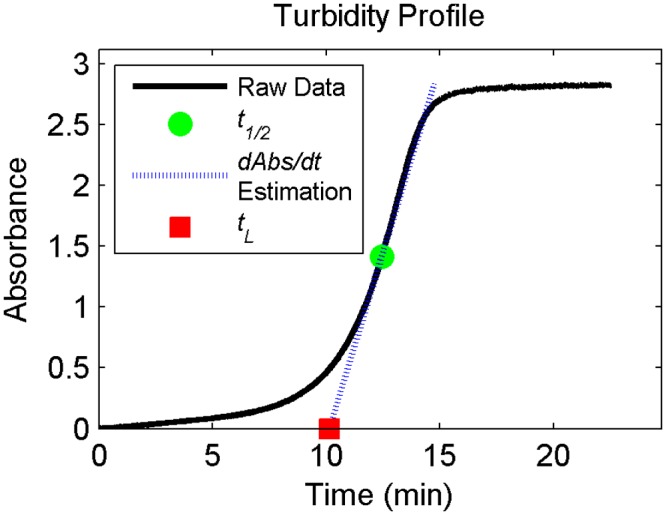
Representative turbidity profile annotated with quantitative metrics. Data shown is for 8 mg/ml collagen polymerized at pH 7.9 and 23°C.

Polymerization kinetics were quantified from spectrophotometric measurements of light absorbance (turbidity) in the sample. Turbidity, which has been shown to correlate with degree of polymerization [9], was recorded throughout hydrogel self-assembly using a Cary 5000 UV-Vis-NIR spectrophotometer (Varian) equipped with a Peltier temperature controller. Absorbance at 405nm, zeroed against a reference sample of deionized water, was recorded every 3.6 seconds for up to 2 hours. Following Kreger et al. [9], four parameters were measured from the absorbance profiles: total change in absorbance *ΔAbs*, polymerization half-time*t*_*1/2*_ (measured at the time at which half the total change in absorbance was achieved), polymerization rate *dAbs/dt* (measured as the slope of the absorbance profile at the half-time), and lag time (*t*_*L*_) (measured as the initial-absorbance intercept of the line intersecting [*t*_*1/2*_, *ΔAbs*] with slope *dAbs/dt*) (Fig 1). Polymerization kinetics were recorded for two to four samples per experimental case.

### Confined Compression

The compression modulus of collagen hydrogels was measured in quasi-steady uniaxial confined compression. This configuration was selected because collagen hydrogels are commonly used to mimic tissues for which relatively long time scales and small strains are physiologically relevant. Furthermore, we assume that the hydrogel is linear elastic under such deformation and that the slope of the stress-strain response represents the compression modulus. Hydrogels were prepared as described in S1 Methods. Before compression, the coverslip was removed from each sample and a drop of distilled water was placed on the surface to ensure hydration during the experiment. Subsequently, a confining nylon plug (8 mm diameter, 6 mm height) was placed on the surface of the hydrogel and the entire sample assembly was placed on the lower platen of a mechanical load frame (Electroforce 3100, Bose) fitted with either a high-resolution low-force transducer (250g_f_, Honeywell) for 4 mg/ml samples or a low-resolution high-force transducer (22 N, Bose) for 6–10 mg/ml samples. The upper platen was lowered approximately to the upper surface of the plug and displaced 1.7 mm at a rate of 0.0085 mm/s. With a sample height of 8.5 mm, this protocol achieved 0.1% strain/s over a range of 0–20% strain. Stress was calculated from the force response divided by the area of the nylon plug (50.3 mm^2^). The radial gap between the nylon plug and sample confinement cylinder (0.75 mm) was designed to permit fluid to escape during confined compression. All measurements were performed at room temperature (23°C); the total duration of each experiment was less than 5 minutes.

**Fig 2:**
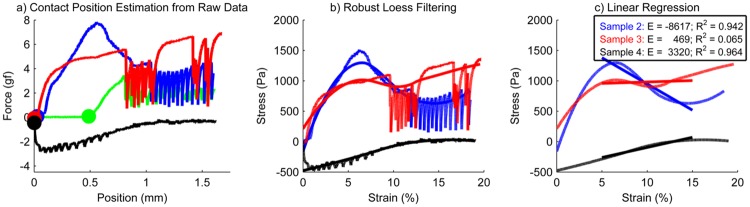
Analysis of compression data from four representative hydrogel samples. All data shown is 4 mg/ml, pH 8.4, 37°C. a) Identification of contact position, marked by a filled circle. b) Robust loess smoothing. c) Linear regression of smoothed data from 5–15% strain. Sample 1 (green) is discarded after step (a) because the sample thickness, determined from the contact position, is 6.9% (585 μm) less than the nominal thickness. Sample 2 (blue) is discarded after step (c) because E = -8617 Pa < 0. Sample 3 (red) is also discarded after step (c) because the regression R^2^ = 0.065 < 0.5. Sample 4 (black) is considered valid and returns a compression modulus of 3320 Pa which contributes to the mean compression modulus calculated for this fabrication condition.

Due to inhomogeneities inherent in collagen hydrogels, escape of bubbles during compression, and imperfections in alignment of system components, stress measurements were not always linear. Therefore, data filtering was necessary to obtain accurate estimates of the compression modulus from slow-displacement measurements of collagen (Fig 2). As this has not been explicitly addressed in previous studies, we have developed a robust algorithm for an automated and fully objective analysis of compression data using MATLAB. First, initial contact of the upper platen with the sample was identified as the position at which the signal from the load cell deviated from the initial value by more than 5 times the sensor RMS noise (Fig 2a). Samples with greater than 5% thickness variation from the nominal value of 8.5 mm (425 μm), as determined by contact position, were discarded. Next, a robust loess-smoothing filter with a large kernel (75% of the data) was applied to minimize the effect of outliers and nonlinearities in the measurements and force/position were converted to compressive stress/compressive strain measurements (Fig 2b). Subsequently, a linear regression was performed on the robust loess-smoothed data between 5–15% strain (Fig 2c). Samples with negative slope were discarded, as physics require that the compression modulus be positive. Finally, poroviscoelastic theory and previous experimental characterization indicate that collagen hydrogel compression modulus is nearly constant between 0 and 20% strain at low displacement rates [11].Samples with strong nonlinearities due to experimental artifacts, identified as those with regression R^2^ below 0.5, were discarded. Experiments were repeated until a minimum of 4 valid samples was obtained for each experimental case.

### Fiber Structure

Confocal reflectance microscopy was used to measure the structure of the collagen network, as it requires no sample preparation or drying and therefore provides accurate dimensional information of samples in the hydrated state [8]. Fiber structure images were acquired using a laser-scanning confocal microscope (LSM 510, Zeiss)configured with a 40X water immersion objective (C-Apochromat, NA = 1.2), pinhole set to 1 Airy unit, and 80/20 beamsplitter to record reflected light from a 543 nm HeNe laser. The image size was set to 112.5 x 112.5μm with a field of view of 2048 x 2048 pixels for a pixel resolution of 54.9 nm. Images were acquired in the center plane of the hydrogels in multi-well slides in order to maximize the distance from artificial boundaries. 12 images were obtained for each experimental case.

**Fig 3:**
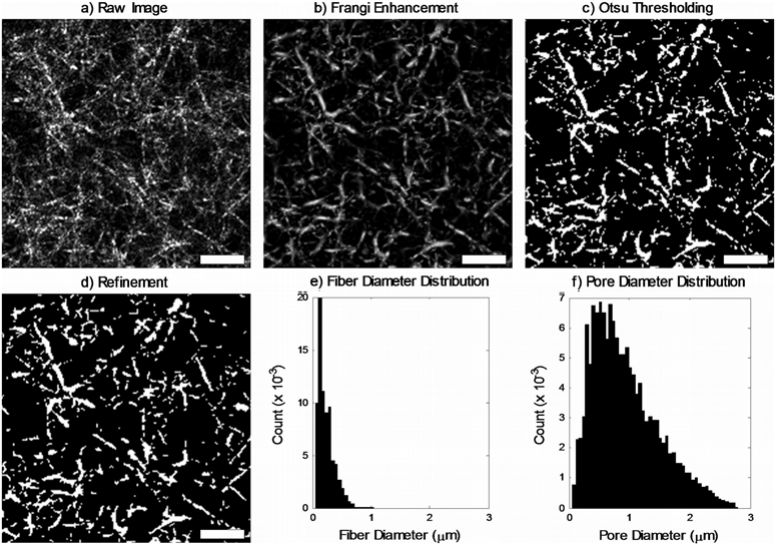
Implementation of fiber analysis. Sample shown is a 1024x1024 pixel subimage of a 4 mg/ml hydrogel polymerized at pH 7.4 and 37°C. Scale bar 10 μm.

In order to accurately evaluate fiber structure images for the relatively high collagen concentrations investigated in this study, an algorithm based on the Frangi vesselness enhancement filter [20] was implemented in MATLAB using the Image Processing Toolbox as well as algorithms available on the MATLAB File Exchange [21,22]. Processing steps are demonstrated in Fig 3, beginning with a subset of a sample raw image (Fig 3a). First, histogram equalization *histeq* was applied to the fiber structure image. Subsequently, the Frangi vesselness filter was applied to enhance fibers (Fig 3b). The parameters for the vesselness filter were selected as follows: the first correction constant, which represents the deviation of the fibers from a blob-like structure, was set to 0.5 as suggested by Frangi [20]. Similarly, the second correction constant, which provides scaling based on the image intensity distribution, was selected as suggested by Frangi to be half the maximum Hessian norm for the image [20]. Finally, the range and step size of vessel scales in the filter kernel were specified based on an expected vessel diameter *D*_*exp*_ of 0.4 μm [9]. The scale range was set to 0.5 *D*_*exp*_—2 *D*_*exp*_with a step size of 0.5 *D*_*exp*_.

After enhancement of in-plane fiber structures using the Frangi vesselness filter, the image was binarized to obtain white fibers on a black background and eliminate low-intensity out-of-plane fibers using Otsu’s method for thresholding (Fig 3c).Subsequently, small objects which were unlikely to represent fibers were removed. The object size threshold for removal was the area corresponding to a circular region with diameter *D*_*exp*_. Finally, morphological artifacts such as holes in fibers and small tendrils were eliminated using *imopen* and *imclose* with a disk-shaped structural element with diameter *D*_*exp*_ /4 (Fig 3d).

To determine fiber size, a distance map of the white regions (fibers) was computed using *bwdist* and skeletonized using *bwmorph* with option *skel*. The remaining pixel values, representing the distance of the central pixel of each fiber from the nearest background pixel, were taken as a measure of fiber radius. The distribution of fiber diameter is shown in Fig 3e. Similarly, pore size was obtained from a distance map of the black regions (pores) followed by a shrink operation using *bwmorph* with option *shrink*. The remaining pixel values, representing the distance of the central pixel of each pore from the nearest fiber pixel, were taken as a measure of pore radius. The distribution of pore diameter is shown in Fig 3f. The mean fiber and pore diameter were used as representative metrics for each image.

### Diffusivity

FRAP is a well-established technique in which a region of a sample containing fluorescent molecules is bleached using a laser-scanning confocal microscope. The subsequent exchange of bleached molecules with fluorescing molecules due to diffusion is imaged and used to calculate the rate of diffusion [5,6,23].Diffusivity measurements using FRAP were acquired at the same sample locations as fiber structure images. The confocal microscope was reconfigured for fluorescence imaging of the FITC-labeled dextran (488 nm beamsplitter/505 nm longpass) with the same 40X water immersion objective and pinhole of 1 Airy unit. The image size was set to 225 x 225μm with a field of view of 512 x 512 pixels for a pixel resolution of 0.439 μm. Images were acquired every 1.5 s using bidirectional scanning to minimize acquisition time. Five pre-bleach images were acquired using a 488nm Argon laser at 1% power in order to obtain a background image. Subsequently, all available lasers and wavelengths (Argon 458/488/514 nm, HeNe 543 nm, HeNe 642 nm) were used at full power to bleach a horizontal region in the center of the sample with a height of 10 pixels and width of 768 pixels. The width of the bleach region was set to 150% of the image width in order to eliminate diffusion edge effects. After bleaching, 5 post-bleach images were recorded using the pre-bleach image settings. FRAP was performed at room temperature of 24 ± 1°C.

Analysis of FRAP images to obtain diffusion rates was performed using the MATLAB-based algorithm developed by Seiffert and Oppermann [24]. Briefly, this algorithm fits a Gaussian distribution to the fluorescent intensity profile in each post-bleach image and estimates the diffusion rate from the decay of the Gaussian over time. Pre-bleach image subtraction was implemented to minimize the effect of background heterogeneity mentioned previously, while ahigh threshold of 0.8 was selected as the maximum acceptable change in Gaussian standard deviation between frames in order to capture the rapid diffusion of 1.5 nm (4 kDa) dextran. FRAP samples with an overall regression R^2^ below 0.9 were discarded. In particular, the diffusion rate for the 1.5 nm dextran was near the limit of what could be captured with the confocal microscope used here, and a significant number of samples were discarded based on this criterion. 6 valid samples were obtained for each experimental case.

### Statistical Analysis

All statistical analysis was performed using JMP software (SAS). A two-way ANOVA was applied to data from each characterization method to identify statistically significant interactions with fabrication parameters. Tukey’s HSD means comparison was used to determine the degree of significance (p-value) of interactions. In all subsequent figures, p-values are denoted with either asterisks (*) or pound symbols (#) as follows: * or #: significant at p<0.05, ** or ##: significant at p<0.01, *** or ###: significant at p<0.001, and **** or ####: significant at p<0.0001. The number of replicates varied depending on the characterization method: N = 2–4 for polymerization kinetics, N = 4–16 for compression modulus, N = 12 for fiber structure, and N = 6 for diffusivity.

Multivariate linear least squares regression was performed on the aggregated characterization data to obtain empirical models of hydrogel properties as a function of fabrication parameters. Because experiments were performed using a full factorial design, fabrication parameters are uncorrelated. For each property (e.g. polymerization half-time), a first ANOVA including all independent variables (concentration, temperature, pH, and hydrodynamic radius if relevant) and first-order interactions was performed. A second ANOVA including only significant interactions was performed in order to obtain the model coefficients. Primary effects were considered significant at p<0.05 while interactions were only considered significant at p<0.01.

### Validation of regression model

In order to validate the empirical model derived from characterization experiments and demonstrate its utility for practical applications, hydrogels were tuned to mimic breast tissue. Target compression moduli were selected to match normal (2000 Pa) and cancerous (4000 Pa) human breast tissue [14,25]. Collagen concentration was fixed at 6 mg/ml, while the readily available temperatures of 23 and 37°C were selected after qualitative verification that the target compression moduli could be achieved under these conditions. Using the Prediction Profiler tool in JMP, the model was optimized to maximize pore diameter and minimize polymerization half-time while targeting desired compression moduli.

Collagen hydrogels were fabricated using the parameters obtained from the model optimization and their properties were characterized using the protocols described above with N = 2 for polymerization kinetics, N = 3 for compression, N = 3 for fiber structure, and N = 6 for diffusivity measurements. In order to investigate the effect, if any, of cells within the gels, 2x10^6^/mL MDA-MB-231 human breast cancer cells (American Type Culture Collection, Manassas VA) were suspended in the buffer solution immediately before incorporation of acidic collagen and initiation of polymerization. Characterization was performed immediately after gel formation as cell proliferation and remodeling may alter hydrogel properties over time. Diffusion measurements were performed using 40 kDa dextran.

## Results

### Polymerization Kinetics

**Fig 4:**
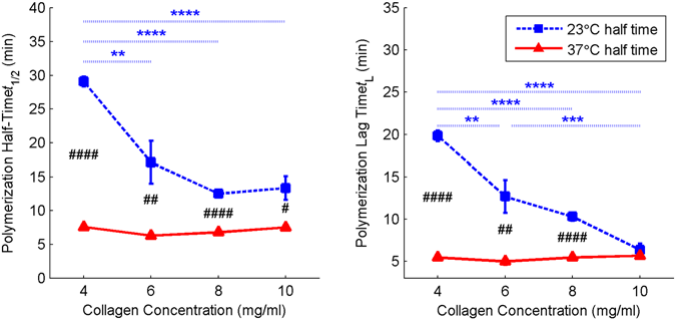
Kinetics of collagen hydrogel polymerization. Blue square symbols with dashed lines represent hydrogels polymerized at 23°C while red triangle symbols with solid lines represent hydrogels polymerized at 37°C. As the effect of pH was statistically non-significant (p>0.05), data is averaged over all three pH groups. Data shown are mean + SE with N = 6–12. Significance was calculated for pH-averaged groups: temperature means comparison is indicated with # while concentration means comparison is indicated with *. At 37°C (solid red lines), concentration had no significant effect on either polymerization half-time or polymerization lag time.

Fig 4 summarizes the mean and standard error (SE) of polymerization half time and lag time across the range of fabrication conditions considered. The p-value of temperature means comparisons is indicated with #. No significant relationships between pH and polymerization kinetics metrics were found (p>0.05) except for total change in absorbance (p<0.0001). All polymerization kinetics data, including polymerization rate and total change in absorbance, are tabulated in S1 Table.

### Compression Modulus

Validation of compression data using the algorithm presented in the methodsresulted in rejection of primarily low-concentration low-temperature gels. Overall, 74% of compression samples were determined to be valid; however, only 52% of 4 mg/ml samples were retained after validation. This is because the sample nonlinearities which necessitate validation are more significant for samples with low compression modulus and therefore low signal than for samples with high compression modulus and high signal.

**Fig 5:**
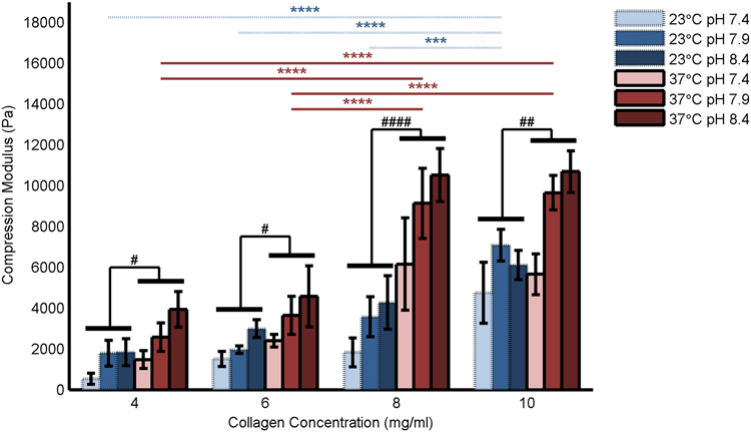
Compression modulus of collagen hydrogels at 0.1%/s deformation rate. Blue bars with dashed lines represent hydrogels polymerized at 23°C while red bars with solid lines represent hydrogels polymerized at 37°C. pH is indicated by color saturation. Data shown are mean + SE with N = 4–16 per bar. Significance was calculated for pH-averaged groups (N = 12–48) as indicated by horizontal black bars. Temperature means comparison is indicated with # while hydrodynamic radius means comparison is indicated with *.

The slope measurements from all valid data sets for each fabrication condition were averaged to obtain the mean compression modulus, with a total of 4 to 16 valid runs for each fabrication condition. The results for all fabrication conditions are shown in Fig 5 and tabulated in S2 Table. For simplicity, pH means comparison and significance are omitted from Fig 5.

### Fiber Structure

**Fig 6:**
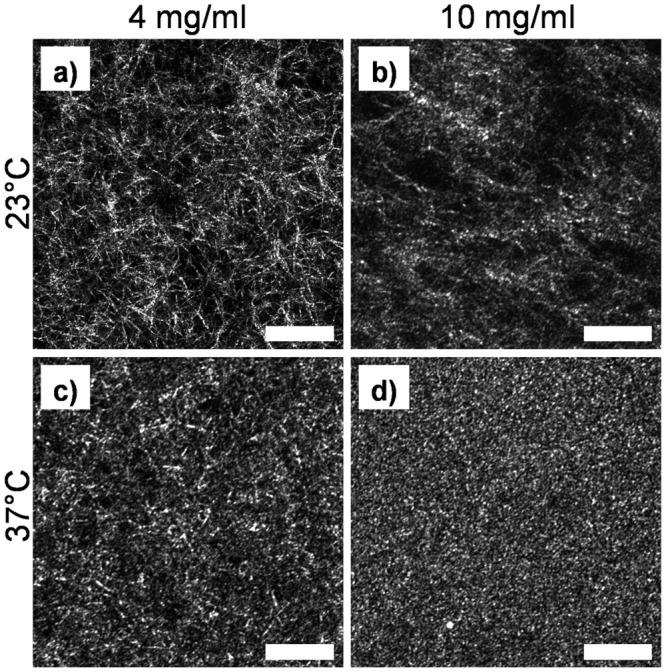
Fiber structure images obtained from confocal reflectance. Images shown are for hydrogels polymerized at pH 7.4. Scale bar 25 μm.

Fiber images obtained using confocal reflectance microscopy were qualitatively similar to those previously reported [19,26,27]. However, our images exhibited somewhat denser, less organized networks because our hydrogels contained higher concentrations of collagen than those imaged in previous studies. Hydrogels polymerized at low temperature exhibited more network-like structures (Fig 6a and 6b), while those polymerized at high temperatures produced more homogeneously distributed fibers with a smaller mesh size (Fig 6c and 6d). High-concentration hydrogels polymerized at low temperature were often very heterogeneous despite careful sample preparation and mixing (Fig 6b). This effect, although still present, decreased at high temperature (Fig 6d). The wide distribution of pore and fiber diameter within a single image can be seen in Fig 3e and 3f. Fibers in reflectance images often appear discontinuous because some reflected light is blocked by near-field (out-of-plane) fibers and does not reach the sensor.

**Fig 7:**
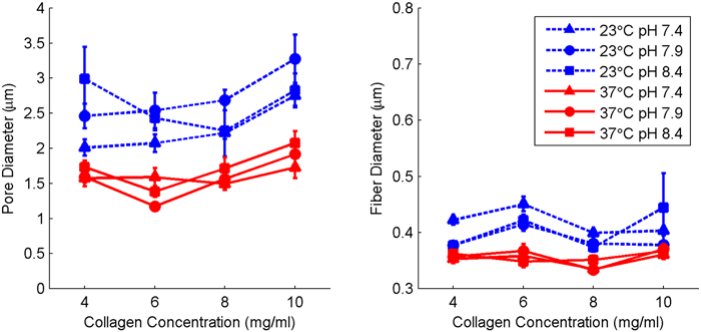
Pore and fiber diameter of collagen hydrogels. Blue symbols with dashed lines represent hydrogels polymerized at 23°C while red symbols with solid lines represent hydrogels polymerized at 37°C. Data shown are mean + SE with N = 12. Significance was calculated for pH-averaged groups. At each concentration, the difference between means at T = 23°C and T = 37°C is significant at p<0.0001 for both pore diameter and fiber diameter.

The results of fiber structure image analysis for all fabrication conditions are shown in Fig 7 and tabulated in S3 Table. While pH and concentration had no significant impact on fiber structure, increasing temperature reduced both pore and fiber diameter significantly (p<0.0001). The mean pore diameter at 23°C was 2.54± 0.37 μm while the mean pore diameter at 37°C was 1.63± 0.23 μm. The temperature dependence of fiber diameter, although statistically significant, had a far smaller magnitude as the mean fiber diameter was 404 ± 26 nm at 23°C and 355 ± 12 nm at 37°C.

### Diffusivity

**Fig 8:**
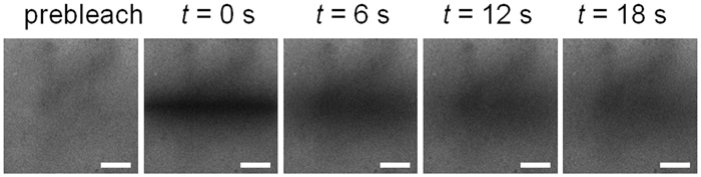
Representative FRAP image sequence. Data shown is for 40 kDa dextran in 10 mg/ml collagen hydrogel polymerized at pH 7.4 and 23°C. Left to right: Prebleach; t = 0 s, t = 6 s, t = 12 s, t = 18 s. Scale bar 50 μm.

Fig 8 shows a sequence of sample images from a FRAP data set. Many data sets (especially those with high MW dextran) contained some heterogeneity in the level of background fluorescence, including the sample shown in Fig 8. However, this did not appear to influence diffusivity measurements, with heterogeneous and homogeneous data sets producing similar results. Diffusion occurred rapidly for all dextrans, as reflected in Fig 8 where high contrast is seen in the bleached region in the first post-bleach image, but little to no contrast remains in the image acquired 18 seconds post-bleach.

**Fig 9:**
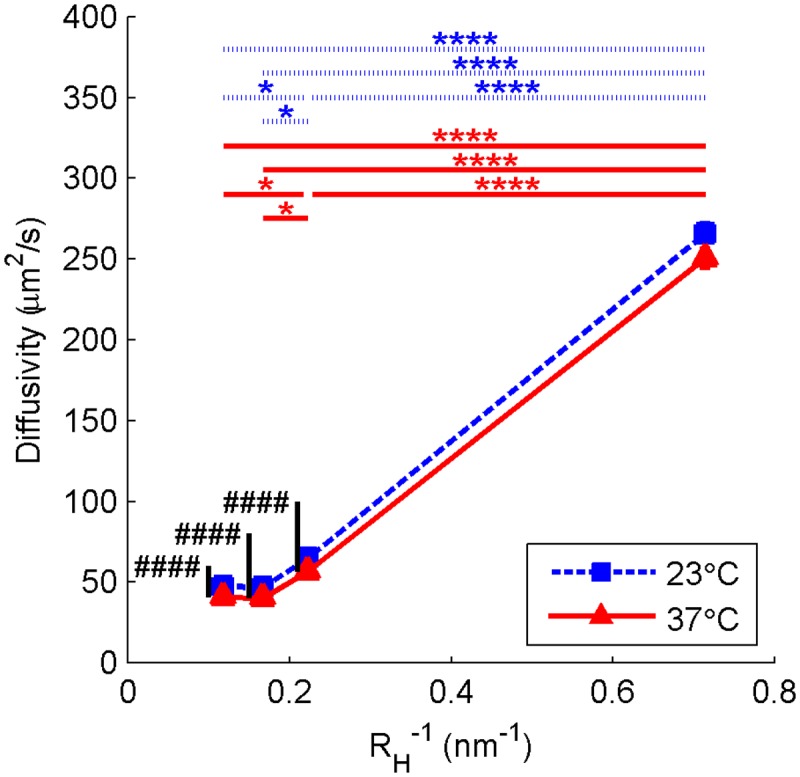
Rate of diffusion of dextran in collagen hydrogels. Blue square symbols with dashed lines represent hydrogels polymerized at 23°C while red triangle symbols with solid lines represent hydrogels polymerized at 37°C. Data shown are mean + SE with N = 72. Each measurement is averaged over pH and concentration. Significance was calculated for pH- and concentration-averaged groups: temperature means comparison is indicated with # while hydrodynamic radius means comparison is indicated with *.

Fig 9 shows the dependence of diffusion rate on dextran size and polymerization temperature (tabulated data, including concentration and pH dependence, can be found in S4 Table). The data are plotted as a function of the inverse of the Stokes (hydrodynamic) radius *R*_*H*_ of the dextrans considered rather than their molecular weight.

## Discussion

The results presented here demonstrate characterization of material properties of collagen I hydrogels over a range of collagen concentration, polymerization temperature, and polymerization pH. The following sections discuss in detail the correlations found between fabrication parameters and material properties. Furthermore, an empirical model and a sensitivity analysis that mathematically describe these correlations are presented.

### Correlations between fabrication parameters and material properties

#### Polymerization Kinetics

Polymerization kinetics data provide temporal metrics which can be used to optimize experimental protocols: for example, knowledge of the polymerization lag-time can be used to determine the maximum time allowable for device assembly, while knowledge of the polymerization half-time can be used to minimize the duration of incubation at temperatures unfavorable for cell viability. Regardless of fabrication conditions, polymerization of all samples followed the characteristic sigmoid function measured as early as 1960 for collagen fibril formation (Fig 1) [28].The time scales of polymerization, specifically half time and lag time, were inversely correlated with polymerization temperature and collagen concentration while the polymerization rate generally increased with temperature and concentration (Fig 4). At the higher temperature (37°C), the correlation with concentration vanishes. This indicates that thermal energy is likely the rate-limiting factor at the low temperature condition, while a different factor (e.g. steric hindrance) becomes the rate-limiting factor at the higher temperature condition. These results are consistent with those published by Hwang and Lyobovitsky [26].

There is some disagreement in the literature with regards to the correlation between pH and polymerization kinetics [10,28,29]. However, differences in other fabrication parameters are likely the cause of discrepancies. Here we report no significant correlation of kinetic metrics with pH; however, this is unsurprising considering the limited range studied here (7.4–8.4). Although increased pH has been reported to correlate with decreased polymerization rate under fabrication conditions similar to those used here, this was for a large pH range (7–10)[10].Over the pH range used in our study, no significant correlation has been previously demonstrated.

Polymerization kinetics measurements and statistics should be interpreted with care as the sample number (N = 2–4) is low. However, even with a limited sample size the resulting measurements are sufficient to provide practical information about polymerization time which may guide design of experiments.

#### Compression Modulus

Unlike polymerization kinetics, which was independent of pH and only dependent on concentration at low temperature, compression modulus was found to increase with all three fabrication parameters. The positive correlation between hydrogel concentration and compression modulus demonstrated here is well-known [9,13,30].However, the relationship between polymerization temperature and compressive modulus has not been well characterized, and previous studies present contradictory findings [4]. The relationship seems to be dependent on pH and concentration ranges, and possibly deformation mode and rate as well. In this study, a strong positive correlation was found between temperature and compressive modulus. We hypothesize that, although no significant interaction between temperature and concentration was found within the ranges examined in this study, the effect of temperature may indeed be enhanced as concentration increases, and that the high absolute concentration studied here, relative to previous studies, enables the conclusive correlation of compression modulus with temperature found herein. The positive correlation of pH with compression modulus found in the present study is supported by previous studies [10,31] and likely due to the effect of stronger intermolecular forces as suggested by Rosenblatt et al. [32].

#### Fiber Structure

In this study, no correlation was found between collagen concentration and fiber diameter, a conclusion supported by previous studies [9,30]. However, in contrast to previous studies [30,33], we measured a weak but statistically significant (p = 0.032 at 23°C, p = 0.0035 at 37°C, pH-averaged) positive correlation between collagen concentration and pore diameter. However, the absolute change in pore diameter was minimal: less than 0.5 μm over an increase of 6 mg/ml collagen content. Both decreased fiber diameter and decreased pore diameter were correlated with increased temperature due to faster precipitation and the presence of more nucleation sites, as demonstrated previously [8,10,15,27,31]. Although previous studies identified a similar relationship with pH [7,10,19],only a very weak correlation was found with pH in this study, likely due to the limited range.

#### Diffusion

The overall inverse relationship between diffusivity of dextrans in collagen hydrogels and their hydrodynamic radii closely matches that predicted by the Stokes-Einstein relation for diffusion of spherical particles in a homogeneous fluid: *D* = (*k*_*B*_ *T*)/(*6πηR*_*H*_), where *k*_*B*_ is Boltzmann’s constant, *T* is temperature (K), and *η* is the kinematic viscosity of the fluid. Surprisingly, the 150 kDa dextran did not continue this trend, but instead diffused at the same rate as the 70 kDa molecule. We note that an interaction between inverse hydrodynamic radius and pH was found by ANOVA to be significant for diffusion(p<0.0001), and hypothesize these may be related phenomena. Because intermolecular forces are strongly pH dependent and molecule shape depends on the balance of those forces, we hypothesize that the hydrodynamic radius of dextran also varies with pH and that the unexpectedly high diffusivity measured for 150 kDa dextran is in fact due to this deviation of R_H_ from the nominal value reported by the manufacturer (a measurement for which the pH was not specified). In support of this hypothesis, FRAP images with 150 kDa dextran in hydrogels polymerized at high pH (8.4) often contained small (several pixel) regions with high fluorescence intensity, indicating a degree of aggregation of the FITC-dextran (data not shown). This phenomenon only occurred for this particular combination of parameters.

The influence of hydrodynamic radius on diffusivity was far stronger than the influence of any hydrogel fabrication parameters, reinforcing previous observations that the hydrogel fiber structure does not significantly impede diffusion of particles with hydrodynamic radius (here: 1.4–8.5 nm) significantly smaller than the pore size of the network (here: 1.6–2.5 μm) [5,23]. However, weak correlations were found between each parameter and diffusion rate. Increasing concentration resulted in reduced diffusivity, similarly to results published by Ramanujan et al. and Erikson et al., who examined concentration ranges of 0–45 mg/ml and 2–20 mg/ml, respectively[5,6].To our knowledge, the relationship between diffusivity and hydrogel polymerization temperature and pH has not been previously measured. Here, we report a statistically significant (p = 0.002) negative correlation between polymerization temperature and diffusivity. We also measure a positive but small relationship between pH and diffusivity. In fact, the effect of pH on diffusivity was ultimately most significant in the interaction with hydrodynamic radius.

### Predictive model of hydrogel properties

The objective of this study was not only to elucidate the relationship between collagen material properties and a broad range of fabrication conditions, but more importantly to generate a set of equations that can be used to optimize fabrication conditions to approximate the properties of a specific *in vivo* tissue. The multivariate linear least squares regression described in an earlier section was performed for polymerization half-time (Eq 1), compression modulus (Eq 2), pore diameter (Eq 3), and diffusivity (Eq 4). Intermediate results from the ANOVA can be found in S5 Table. Regression equations and ANOVA results for other parameters can be provided upon request.

$$t_{1/2}=21.89-12.69C^{\prime }-17.45T^{\prime }+13.77C^{\prime }T^{\prime }\text{​}$$ (1)

$$E=-853+5725C^{\prime }+2720T^{\prime }+2241pH^{\prime }\text{​}$$ (2)

$$\theta_{P}=2.236+0.369C^{\prime }-0.915T^{\prime }+0.245pH^{\prime }\text{​}$$ (3)

$$D=49.02-21.1C^{\prime }-9.33T^{\prime }-5.73pH^{\prime }+190.23{\left(R_{H}^{-1}\right)}^{\prime }+0.37pH\cdot \text{​}{\left(R_{H}^{-1}\right)}^{\prime }$$ (4)

where *t*_*1/2*_ is in minutes, *E* is in Pa, *Ø*_*P*_ is in μm, and *D* is in μm^2^/s. *C*, *T*, *pH*, and *R*_*H*_^*-1*^ have been transformed from the experimental domain to non-dimensional parameters *C′*, *T*′, *pH′* and *(R*_*H*_^*-1*^ *)′*, each spanning a domain of 0–1 in order to enable comparison (Eqs 5–8):

$$C^{\prime }=\frac{C-C_{MIN}}{C_{MAX}-C_{MIN}}=\frac{C-4}{6}$$ (5)

$$T^{\prime }=\frac{T-T_{MIN}}{T_{MAX}-T_{MIN}}=\frac{T-23}{14}$$ (6)

$$pH^{\prime }=\frac{pH-pH_{MIN}}{pH_{MAX}-pH_{MIN}}=pH-7.4$$ (7)

$${\left(R_{H}^{-1}\right)}^{\prime }=\frac{R_{H}^{-1}-{\left(R_{H,MAX}\right)}^{-1}}{{\left(R_{H,MIN}\right)}^{-1}-{\left(R_{H,MAX}\right)}^{-1}}=\frac{R_{H}^{-1}-0.118}{0.597}$$ (8)

where *C* is in mg/ml, *T* is in °C, *pH* is unitless, *R*_*H*_ is in nm, and *MIN* and *MAX* denote the extents of the experimental ranges (4–10 mg/ml, 23–37°C, 7.4–8.4, and 1.4–8.5 nm, respectively). It should be noted that, if the equations are used as a predictive model to estimate the required fabrication condition to obtain desired properties, it remains strictly valid only within the experimental ranges, i.e. where the nondimensionalized fabrication parameters remain between 0 and 1. Values outside this range represent extrapolation of the data and should be used with caution.

Using the full set of raw data, higher-order optimization can be performed using functions available in statistical software. For example, the Prediction Profiler in JMP (SAS) can be applied to the multivariate data set in conjunction with a set of desirability functions in order to find a fabrication condition which maximizes overall desirability. For the reader interested in performing such analysis, the raw data set can be provided upon request.

A sensitivity table demonstrating the relative influence of each fabrication parameter and interaction term on each material property was derived from these equations (Table 2). By normalizing to the largest coefficient, the utility of each parameter for control of each property is elucidated. Here, the relationships examined in detail earlier in the discussion can be easily seen. For instance, the lack of correlation between polymerization half-time and pH contrasted with the strength of the interaction between concentration and temperature, which is comparable to the concentration term and nearly as significant as the temperature term. The intent of this table is use as a quick guide for preliminary design of fabrication parameters for collagen hydrogels.

**Table 2:** Sensitivity of key hydrogel material properties to fabrication parameters. Sensitivities are obtained from the multiple regression coefficients, normalized to the largest coefficient in each model. The R^2^ of the multivariate fit is indicated for each property. Analysis was performed on transformed (nondimensionalized) parameters. Only factors with p<0.01 (ANOVA) are shown.

	*t*_*1/2*_(R^2^ = 0.565)				*E* (R^2^ = 0.504)				*Ø*_P_ (R^2^ = 0.332)				*D* (R^2^ = 0.867)			
	***C'***	***T'***	***pH'***		***C'***	***T'***	***pH'***		***C'***	***T'***	***pH'***		***C'***	***T'***	***pH'***	***(r***_*H*_^*-1*^ ***)'***
***C'***	-0.78	0.79	-	***C'***	1	-	-	***C'***	0.40	-	-	***C'***	-0.11	-	-	-
***T'***	0.79	-1	-	***T'***	-	0.48	-	***T'***	-	-1	-	***T'***	-	-0.05	-	-
***pH'***	-	-	-	***pH'***	-	-	0.39	***pH'***	-	-	0.27	***pH'***	-	-	-0.03	0.37
												***(r***_***H***_^***-1***^ ***)'***	-	-	0.37	1

### Proof-of-concept for tissue engineering using tunable hydrogels

**Fig 10:**
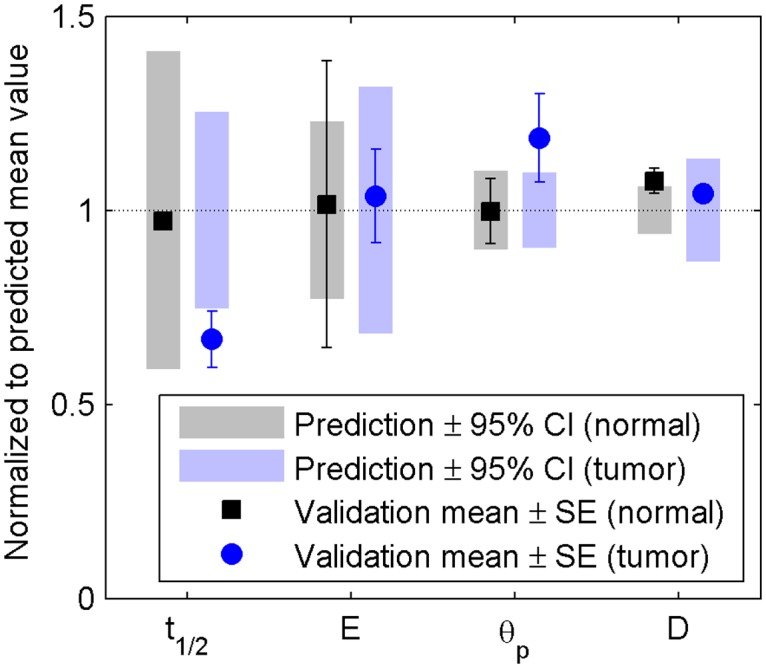
Validation of model for design of tuned hydrogels. Each parameter is normalized to the target value (predicted mean). Symbols with error bars represent the mean and standard error of the validation data set. Shaded bars indicate the upper and lower 95% confidence interval on the predicted mean value, obtained from JMP. The validation set targeted to mimic normal breast tissue is plotted in gray/black (squares) while the set targeted to mimic cancerous breast tissue is plotted in blue (circles).

As shown in Fig 10, the properties of the validation set containing cells match closely with the model predictions, with only one instance (polymerization half-time, tumor mimic, N = 2) in which the standard error on the mean of the validation data is slightly outside the 95% confidence interval on the predicted mean.

These data indicate that the empirical model accurately predicts hydrogel properties and can be used for tuning collagen hydrogels for *in vitro* applications. Although the presence of cells can change hydrogel properties over time due to degradation, remodeling, and protein synthesis, the properties predicted by the empirical model accurately reflect initial conditions for cell-containing hydrogels.

## Implications for Design of Hydrogels

This work has quantitatively measured the sensitivity of a variety of collagen hydrogel material properties, including compression modulus, pore size, and diffusivity, to fabrication parameters varied within biological ranges. Although previous studies have suggested that a wide pH range must be used in order to significantly affect hydrogel properties, the results presented here reveal that even the limited range acceptable for viability of cells embedded pre-polymerization has a statistically significant influence on compression modulus and fiber structure. The characterization equations given here provide good coverage of property values within the domain examined. Compression moduli from 540 Pa to 10700 Pa and pore sizes from 1.2 to 3.2 μm can be obtained through optimization of all three parameters. While these ranges are not as large as those found *in vivo*, this represents the broadest range of material properties demonstrated to be feasible for a single type of collagen hydrogel.

For the first time, we report here the sensitivity of hydrogel properties to multiple fabrication parameters. Although diffusivity depends most significantly on molecule size and is not strongly influenced by hydrogel preparation, the other properties measured here show significant correlation with multiple fabrication parameters. We expect that the results of this study will serve as an invaluable tool for tissue engineers across a range of applications, and hope that it will aid in standardization of hydrogel protocols between research groups.

## Supporting Information

S1 Fig Hydrogel configurations for characterization. a) capped cuvette for spectrophotometric kinetics measurements, b) cylindrical mold for confined compression measurements (confining plug not shown), c) multi-well slide for fiber structure and diffusion measurements. Samples shown are 4 mg/ml hydrogels polymerized at pH 8.4 and 37°C.
(TIF)

S1 Methods Preparation of collagen hydrogels. (DOCX)

S1 Table Polymerization kinetics metrics. (DOCX)

S2 Table Compression modulus characterization data. (DOCX)

S3 Table Fiber structure metrics. (DOCX)

S4 Table Diffusivity characterization data. (DOCX)

S5 Table ANOVA results for polymerization half-time, compression modulus, pore diameter, and diffusivity. (DOCX)
