# Supplementary figures and images for: Tunable Collagen I Hydrogels for Engineered Physiological Tissue Micro-Environments
Source: PLoS One. 2015 Mar 30;10(3):e0122500. doi: 10.1371/journal.pone.0122500 (PMC4378848; doi:10.1371/journal.pone.0122500)

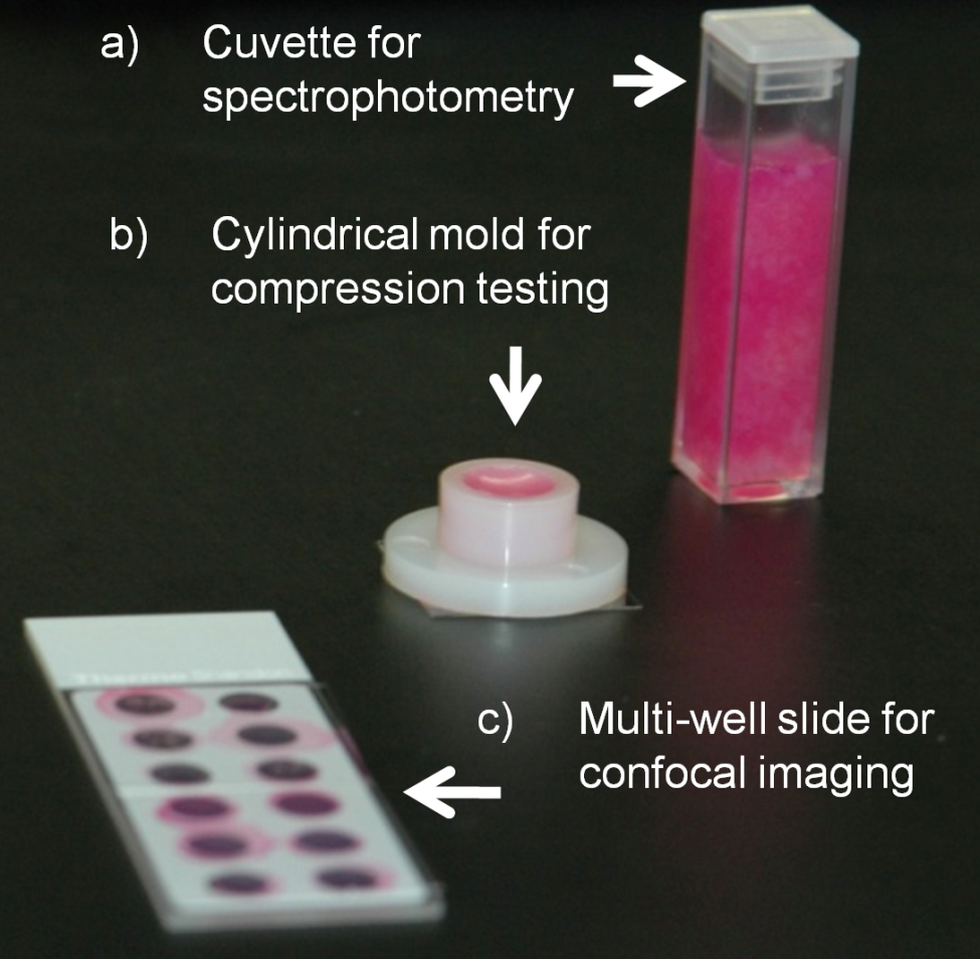

Supplement: S1 Fig — a) capped cuvette for spectrophotometric kinetics measurements, b) cylindrical mold for confined compression measurements (confining plug not shown), c) multi-well slide for fiber structure and diffusion measurements. Samples shown are 4 mg/ml hydrogels polymerized at pH 8.4 and 37°C. (TIF) [file pone.0122500.s001.tif]
