# Supplementary material for: Tunable Collagen I Hydrogels for Engineered Physiological Tissue Micro-Environments
Source: PLoS One. 2015 Mar 30;10(3):e0122500. doi: 10.1371/journal.pone.0122500 (PMC4378848; doi:10.1371/journal.pone.0122500)
Supplement: S1 Methods — (DOCX) [file pone.0122500.s002.docx]

**Methods S1.** Preparation of collagen hydrogels.

Type I collagen was acid-extracted as follows: Tendons removed from rat tails obtained from young male Sprague-Dawley rats (RAT216154-216228, BioreclamationIVT) were added to 0.01 M (pH 2.0) hydrochloric acid (HCl, Sigma) at 4°C, with a volume of approximately 30 mL/tail. The solution was placed in an ice bath and stirred overnight using a magnetic stir plate. Subsequently, the solution was centrifuged at 30,000 *g* for 45 minutes to remove insoluble components. The supernatant was frozen and lyophilized to obtain collagen protein. Lyophilized collagen was stored up to 6 months at -20°C. For reconstitution, a mass of lyophilized collagen was weighed and added to 0.01 M HCl to achieve solution concentrations of 8, 12, 16, and 20 mg/ml. Collagen and acid were mixed, allowed to reconstitute overnight at 4°C, mixed again, and centrifuged for 60 seconds at 1800 *g* to remove bubbles. Collagen solutions were stored up to 6 weeks at 4°C.

Hydrogels were prepared by combining acidic collagen with concentrated cell culture medium (10X Dulbecco’s Modified Eagle’s Medium (DMEM), Sigma D2429), buffered cell culture medium (1X DMEM supplemented with glucose, sodium bicarbonate, and L-glutamine, Sigma D6046), and neutralization agent (1N sodium hydroxide (NaOH), Fisher) according to the following recipe (by volume): 0.1 units 10X DMEM, 0.5 units collagen solution, *k_NaOH_* units 1N NaOH (dependent on desired pH and absolute collagen concentration, Equation 1), remaining units 1X DMEM. To obtain consistent hydrogel pH, a collagen solution with twice the final hydrogel concentration was used. A calibration formula for pH was obtained by adjusting absolute collagen concentration (maintaining the 2:1 dilution ratio) and NaOH fraction in hydrogels and measuring pH of the resulting solution:

|  |  | Equation 1 |
| --- | --- | --- |

where *k_NaOH_* is the volume fraction of 1N NaOH in the hydrogel and *C* is the collagen concentration in the hydrogel, in mg/ml. This calibration formula was developed and tested for pH-controllable hydrogels using the constituents listed; its applicability to alternate hydrogel preparations (using acetic acid-solubilized collagen, for instance) is not known.

For fabrication of all hydrogels, 10X DMEM, 1X DMEM, and 1N NaOH were mixed over ice. After the acidic collagen was added, the solution was mixed using a spatula, taking care to avoid bubble formation. The hydrogel was transferred to an application-specific mold (Figure S2) and incubated at either 23°C or 37°C for a duration at least twice the polymerization half-time as determined by kinetics measurements.

**Figure S2**

Hydrogels for polymerization kinetics measurements were prepared directly in polystyrene spectrophotometric cuvettes (Schaumaplast 10 mm lightpath, Fisher) (Figure S2a). After mixing, cuvettes were capped, placed into a spectrophotometer sample block preset to the desired incubation temperature with a Peltier device, and data recording begun immediately.

Hydrogels for confined compression experiments were prepared in 15 mL centrifuge tubes. After mixing, 600 µL volumes were dispensed using a syringe into cylindrical molds (Ø 9.5 mm, height 9.35 mm), sealed with coverslips to prevent evaporation, and polymerized in an incubator set to the desired temperature. The resulting cylindrical hydrogels had a diameter of 9.5 mm and height 8.5 mm (Figure S2b).

For confocal imaging, solutions were prepared similarly to those for compression tests but pipetted into multi-well slides (Shandon Multi-Spot Slides, Thermo) to obtain hydrogels approximately 100 µm thick with a diameter of 6 mm (Figure S2c). Furthermore, during solution preparation, a portion of the 1X DMEM (1% of the total hydrogel volume) was replaced with an aqueous dextran solution (50 mg/ml for 1.4, 4.5, and 6.0 nm dextrans and 40 mg/ml for 8.5 nm dextran) for a final dextran concentration in the hydrogel of 0.4-0.5 mg/ml. The Stokes’ radii *R_H_* for FITC-dextrans (1.4, 4.5, 6.0 and 8.5 nm for 4, 40, 70, 150 kDa), which scale nearly linearly with the square root of the molecular weight, were obtained from the supplier (Sigma Product Information Sheet FD-4).

Fabrication parameters which can affect hydrogel properties but were not varied in this study were selected to match those most prevalent in the literature. They include collagen source and solubilization (hydrochloric acid-extracted rat tail tendon), solution components (DMEM and NaOH), and ionic strength (~130 mM). The numerical data presented in this study should be interpreted in the context of these fixed parameters.
