# Supplementary material for: Tunable Collagen I Hydrogels for Engineered Physiological Tissue Micro-Environments
Source: PLoS One. 2015 Mar 30;10(3):e0122500. doi: 10.1371/journal.pone.0122500 (PMC4378848; doi:10.1371/journal.pone.0122500)
Supplement: S1 Table — (DOCX) [file pone.0122500.s003.docx]

**S1 Table.** Polymerization kinetics metrics (mean ± SE).

| Concentration (mg/ml) | Polymerization Temperature (°C) | Polymerization pH | Polymerization Half-Time (min) | Polymerization Lag Time (min) | Polymerization Rate (min^-1^) | Total Change in Absorbance |
| --- | --- | --- | --- | --- | --- | --- |
| 4 | 23 | 7.4 | 30.05 ± 1.86 | 19.78 ± 1.89 | 0.085 ± 0.008 | 1.75 ± 0.17 |
| 4 | 23 | 7.9 | 27.90 ± 0.43 | 19.17 ± 0.33 | 0.089 ± 0.002 | 1.56 ± 0.06 |
| 4 | 23 | 8.4 | 29.30 ± 1.32 | 20.53 ± 1.10 | 0.080 ± 0.001 | 1.40 ± 0.03 |
| 4 | 37 | 7.4 | 7.32 ± 0.06 | 5.13 ± 0.29 | 0.337 ± 0.006 | 1.47 ± 0.18 |
| 4 | 37 | 7.9 | 7.41 ± 0.18 | 5.42 ± 0.20 | 0.326 ± 0.003 | 1.30 ± 0.02 |
| 4 | 37 | 8.4 | 8.02 ± 0.65 | 5.82 ± 0.30 | 0.306 ± 0.038 | 1.32 ± 0.04 |
| 6 | 23 | 7.4 | 19.80 ± 6.93 | 14.23 ± 4.18 | 0.426 ± 0.167 | 2.97 ± 0.22 |
| 6 | 23 | 7.9 | 20.12 ± 6.04 | 14.41 ± 3.77 | 0.460 ± 0.197 | 2.54 ± 0.27 |
| 6 | 23 | 8.4 | 11.42 ± 1.46 | 9.43 ± 1.35 | 0.639 ± 0.071 | 2.48 ± 0.03 |
| 6 | 37 | 7.4 | 7.05 ± 0.37 | 5.49 ± 0.09 | 0.896 ± 0.391 | 2.44 ± 0.40 |
| 6 | 37 | 7.9 | 5.37 ± 1.05 | 4.23 ± 1.05 | 1.199 ± 0.107 | 2.71 ± 0.24 |
| 6 | 37 | 8.4 | 6.48 ± 0.74 | 5.24 ± 0.69 | 0.933 ± 0.062 | 2.31 ± 0.06 |
| 8 | 23 | 7.4 | 12.22 ± 0.22 | 9.32 ± 0.57 | 0.571 ± 0.064 | 3.16 ± 0.15 |
| 8 | 23 | 7.9 | 11.89 ± 1.06 | 10.52 ± 0.81 | 0.635 ± 0.076 | 2.83 ± 0.10 |
| 8 | 23 | 8.4 | 13.46 ± 0.99 | 11.09 ± 0.99 | 0.558 ± 0.054 | 2.60 ± 0.13 |
| 8 | 37 | 7.4 | 6.76 ± 0.21 | 5.58 ± 0.17 | 1.265 ± 0.150 | 2.98 ± 0.25 |
| 8 | 37 | 7.9 | 6.16 ± 0.85 | 4.86 ± 0.85 | 0.969 ± 0.019 | 2.51 ± 0.04 |
| 8 | 37 | 8.4 | 7.46 ± 0.46 | 5.94 ± 0.42 | 0.830 ± 0.037 | 2.51 ± 0.05 |
| 10 | 23 | 7.4 | 12.04 ± 2.61 | 7.95 ± 1.42 | 0.467 ± 0.099 | 3.02 ± 0.12 |
| 10 | 23 | 7.9 | 12.63 ± 2.91 | 5.91 ± 1.12 | 0.320 ± 0.114 | 2.78 ± 0.09 |
| 10 | 23 | 8.4 | 15.37 ± 3.93 | 5.26 ± 1.19 | 0.218 ± 0.085 | 2.51 ± 0.04 |
| 10 | 37 | 7.4 | 7.16 ± 1.08 | 5.45 ± 0.35 | 0.819 ± 0.365 | 2.25 ± 0.04 |
| 10 | 37 | 7.9 | 7.92 ± 0.38 | 5.94 ± 0.12 | 0.511 ± 0.037 | 2.00 ± 0.12 |
| 10 | 37 | 8.4 | 7.51 ± 0.53 | 5.58 ± 0.19 | 0.570 ± 0.146 | 2.10 ± 0.18 |
