# Supplementary material for: Tunable Collagen I Hydrogels for Engineered Physiological Tissue Micro-Environments
Source: PLoS One. 2015 Mar 30;10(3):e0122500. doi: 10.1371/journal.pone.0122500 (PMC4378848; doi:10.1371/journal.pone.0122500)
Supplement: S2 Table — (DOCX) [file pone.0122500.s004.docx]

**S2 Table.** Compression modulus characterization data (mean ± SE).

| Concentration (mg/ml) | Polymerization Temperature (°C) | Polymerization pH | Compression Modulus (Pa) |
| --- | --- | --- | --- |
| 4 | 23 | 7.4 | 538 ± 270 |
| 4 | 23 | 7.9 | 1794 ± 634 |
| 4 | 23 | 8.4 | 1834 ± 666 |
| 4 | 37 | 7.4 | 1473 ± 432 |
| 4 | 37 | 7.9 | 2576 ± 700 |
| 4 | 37 | 8.4 | 3933 ± 875 |
| 6 | 23 | 7.4 | 1501 ± 368 |
| 6 | 23 | 7.9 | 1954 ± 186 |
| 6 | 23 | 8.4 | 2991 ± 446 |
| 6 | 37 | 7.4 | 2399 ± 303 |
| 6 | 37 | 7.9 | 3641 ± 940 |
| 6 | 37 | 8.4 | 4578 ± 1498 |
| 8 | 23 | 7.4 | 1826 ± 700 |
| 8 | 23 | 7.9 | 3569 ± 985 |
| 8 | 23 | 8.4 | 4269 ± 1309 |
| 8 | 37 | 7.4 | 6158 ± 2256 |
| 8 | 37 | 7.9 | 9134 ± 1725 |
| 8 | 37 | 8.4 | 10517 ± 1298 |
| 10 | 23 | 7.4 | 4747 ± 1499 |
| 10 | 23 | 7.9 | 7088 ± 779 |
| 10 | 23 | 8.4 | 6111 ± 718 |
| 10 | 37 | 7.4 | 5655 ± 1001 |
| 10 | 37 | 7.9 | 9652 ± 846 |
| 10 | 37 | 8.4 | 10691 ± 1020 |
