# Supplementary material for: Tunable Collagen I Hydrogels for Engineered Physiological Tissue Micro-Environments
Source: PLoS One. 2015 Mar 30;10(3):e0122500. doi: 10.1371/journal.pone.0122500 (PMC4378848; doi:10.1371/journal.pone.0122500)
Supplement: S3 Table — (DOCX) [file pone.0122500.s005.docx]

**S3 Table.** Fiber structure metrics (mean ± SE).

| Concentration (mg/ml) | Polymerization Temperature (°C) | Polymerization pH | Fiber Diameter (µm) | Pore Diameter (µm) |
| --- | --- | --- | --- | --- |
| 4 | 23 | 7.4 | 0.422 ± 0.009 | 2.01 ± 0.12 |
| 4 | 23 | 7.9 | 0.377 ± 0.006 | 2.46 ± 0.17 |
| 4 | 23 | 8.4 | 0.378 ± 0.006 | 2.99 ± 0.46 |
| 4 | 37 | 7.4 | 0.353 ± 0.008 | 1.57 ± 0.11 |
| 4 | 37 | 7.9 | 0.357 ± 0.005 | 1.59 ± 0.08 |
| 4 | 37 | 8.4 | 0.362 ± 0.004 | 1.73 ± 0.09 |
| 6 | 23 | 7.4 | 0.451 ± 0.013 | 2.07 ± 0.12 |
| 6 | 23 | 7.9 | 0.415 ± 0.012 | 2.54 ± 0.26 |
| 6 | 23 | 8.4 | 0.422 ± 0.010 | 2.43 ± 0.17 |
| 6 | 37 | 7.4 | 0.358 ± 0.007 | 1.59 ± 0.13 |
| 6 | 37 | 7.9 | 0.367 ± 0.012 | 1.17 ± 0.03 |
| 6 | 37 | 8.4 | 0.348 ± 0.010 | 1.38 ± 0.08 |
| 8 | 23 | 7.4 | 0.399 ± 0.007 | 2.22 ± 0.09 |
| 8 | 23 | 7.9 | 0.381 ± 0.006 | 2.69 ± 0.15 |
| 8 | 23 | 8.4 | 0.374 ± 0.008 | 2.25 ± 0.37 |
| 8 | 37 | 7.4 | 0.335 ± 0.004 | 1.49 ± 0.09 |
| 8 | 37 | 7.9 | 0.333 ± 0.004 | 1.56 ± 0.15 |
| 8 | 37 | 8.4 | 0.351 ± 0.008 | 1.71 ± 0.16 |
| 10 | 23 | 7.4 | 0.403 ± 0.008 | 2.75 ± 0.15 |
| 10 | 23 | 7.9 | 0.378 ± 0.002 | 3.28 ± 0.34 |
| 10 | 23 | 8.4 | 0.445 ± 0.608 | 2.83 ± 0.24 |
| 10 | 37 | 7.4 | 0.361 ± 0.008 | 1.73 ± 0.16 |
| 10 | 37 | 7.9 | 0.370 ± 0.009 | 1.92 ± 0.16 |
| 10 | 37 | 8.4 | 0.367 ± 0.007 | 2.07 ± 0.17 |
