# Supplementary material for: Tunable Collagen I Hydrogels for Engineered Physiological Tissue Micro-Environments
Source: PLoS One. 2015 Mar 30;10(3):e0122500. doi: 10.1371/journal.pone.0122500 (PMC4378848; doi:10.1371/journal.pone.0122500)
Supplement: S4 Table — (DOCX) [file pone.0122500.s006.docx]

**S4 Table.** Diffusivity characterization data (mean ± SE).

| Dextran MW (kDa) | Stokes (Hydrodynamic) Radius (nm) | Concentration (mg/ml) | Polymerization Temperature (°C) | Polymerization pH | Diffusivity (µm^2^/s) |
| --- | --- | --- | --- | --- | --- |
| 4 | 1.4 | 4 | 23 | 7.4 | 220 ± 14 |
| 4 | 1.4 | 4 | 23 | 7.9 | 302 ± 19 |
| 4 | 1.4 | 4 | 23 | 8.4 | 387 ± 29 |
| 4 | 1.4 | 4 | 37 | 7.4 | 234 ± 21 |
| 4 | 1.4 | 4 | 37 | 7.9 | 277 ± 9 |
| 4 | 1.4 | 4 | 37 | 8.4 | 324 ± 14 |
| 4 | 1.4 | 6 | 23 | 7.4 | 202 ± 9 |
| 4 | 1.4 | 6 | 23 | 7.9 | 252 ± 8 |
| 4 | 1.4 | 6 | 23 | 8.4 | 225 ± 11 |
| 4 | 1.4 | 6 | 37 | 7.4 | 232 ± 7 |
| 4 | 1.4 | 6 | 37 | 7.9 | 233 ± 42 |
| 4 | 1.4 | 6 | 37 | 8.4 | 236 ± 19 |
| 4 | 1.4 | 8 | 23 | 7.4 | 255 ± 14 |
| 4 | 1.4 | 8 | 23 | 7.9 | 313 ± 19 |
| 4 | 1.4 | 8 | 23 | 8.4 | 262 ± 62 |
| 4 | 1.4 | 8 | 37 | 7.4 | 232 ± 15 |
| 4 | 1.4 | 8 | 37 | 7.9 | 293 ± 32 |
| 4 | 1.4 | 8 | 37 | 8.4 | 324 ± 33 |
| 4 | 1.4 | 10 | 23 | 7.4 | 233 ± 28 |
| 4 | 1.4 | 10 | 23 | 7.9 | 276 ± 31 |
| 4 | 1.4 | 10 | 23 | 8.4 | 267 ± 26 |
| 4 | 1.4 | 10 | 37 | 7.4 | 169 ± 9 |
| 4 | 1.4 | 10 | 37 | 7.9 | 185 ± 11 |
| 4 | 1.4 | 10 | 37 | 8.4 | 269 ± 43 |

**S4 Table.** (continued). Diffusivity characterization data (mean ± SE).

| Dextran MW (kDa) | Stokes (Hydrodynamic) Radius (nm) | Concentration (mg/ml) | Polymerization Temperature (°C) | Polymerization pH | Diffusivity (µm^2^/s) |
| --- | --- | --- | --- | --- | --- |
| 40 | 4.5 | 4 | 23 | 7.4 | 69.2 ± 1.8 |
| 40 | 4.5 | 4 | 23 | 7.9 | 67.1 ± 2.3 |
| 40 | 4.5 | 4 | 23 | 8.4 | 72.2 ± 1.5 |
| 40 | 4.5 | 4 | 37 | 7.4 | 62.6 ± 3.1 |
| 40 | 4.5 | 4 | 37 | 7.9 | 61.1 ± 2.1 |
| 40 | 4.5 | 4 | 37 | 8.4 | 57.6 ± 0.7 |
| 40 | 4.5 | 6 | 23 | 7.4 | 71.4 ± 2.0 |
| 40 | 4.5 | 6 | 23 | 7.9 | 75.3 ± 1.1 |
| 40 | 4.5 | 6 | 23 | 8.4 | 77.8 ± 4.8 |
| 40 | 4.5 | 6 | 37 | 7.4 | 69.7 ± 2.4 |
| 40 | 4.5 | 6 | 37 | 7.9 | 62.5 ± 2.0 |
| 40 | 4.5 | 6 | 37 | 8.4 | 74.4 ± 3.2 |
| 40 | 4.5 | 8 | 23 | 7.4 | 59.2 ± 1.3 |
| 40 | 4.5 | 8 | 23 | 7.9 | 67.4 ± 2.5 |
| 40 | 4.5 | 8 | 23 | 8.4 | 63.4 ± 1.7 |
| 40 | 4.5 | 8 | 37 | 7.4 | 41.7 ± 1.6 |
| 40 | 4.5 | 8 | 37 | 7.9 | 50.2 ± 1.7 |
| 40 | 4.5 | 8 | 37 | 8.4 | 63.9 ± 3.0 |
| 40 | 4.5 | 10 | 23 | 7.4 | 55.7 ± 2.0 |
| 40 | 4.5 | 10 | 23 | 7.9 | 48.9 ± 1.3 |
| 40 | 4.5 | 10 | 23 | 8.4 | 51.0 ± 0.9 |
| 40 | 4.5 | 10 | 37 | 7.4 | 46.1 ± 2.5 |
| 40 | 4.5 | 10 | 37 | 7.9 | 44.3 ± 1.8 |
| 40 | 4.5 | 10 | 37 | 8.4 | 47.0 ± 1.1 |

**Table S6** (continued). Diffusivity characterization data (mean ± SE).

| Dextran MW (kDa) | Stokes (Hydrodynamic) Radius (nm) | Concentration (mg/ml) | Polymerization Temperature (°C) | Polymerization pH | Diffusivity (µm^2^/s) |
| --- | --- | --- | --- | --- | --- |
| 70 | 6.0 | 4 | 23 | 7.4 | 50.5 ± 1.6 |
| 70 | 6.0 | 4 | 23 | 7.9 | 57.4 ± 5.7 |
| 70 | 6.0 | 4 | 23 | 8.4 | 51.2 ± 4.0 |
| 70 | 6.0 | 4 | 37 | 7.4 | 50.7 ± 1.3 |
| 70 | 6.0 | 4 | 37 | 7.9 | 46.7 ± 1.4 |
| 70 | 6.0 | 4 | 37 | 8.4 | 50.8 ± 5.2 |
| 70 | 6.0 | 6 | 23 | 7.4 | 45.0 ± 0.4 |
| 70 | 6.0 | 6 | 23 | 7.9 | 49.1 ± 2.8 |
| 70 | 6.0 | 6 | 23 | 8.4 | 40.9 ± 0.6 |
| 70 | 6.0 | 6 | 37 | 7.4 | 45.6 ± 1.6 |
| 70 | 6.0 | 6 | 37 | 7.9 | 29.2 ± 0.3 |
| 70 | 6.0 | 6 | 37 | 8.4 | 41.9 ± 2.8 |
| 70 | 6.0 | 8 | 23 | 7.4 | 53.9 ± 1.4 |
| 70 | 6.0 | 8 | 23 | 7.9 | 55.5 ± 0.7 |
| 70 | 6.0 | 8 | 23 | 8.4 | 48.7 ± 1.3 |
| 70 | 6.0 | 8 | 37 | 7.4 | 38.4 ± 1.0 |
| 70 | 6.0 | 8 | 37 | 7.9 | 39.4 ± 2.6 |
| 70 | 6.0 | 8 | 37 | 8.4 | 50.4 ± 1.8 |
| 70 | 6.0 | 10 | 23 | 7.4 | 38.7 ± 2.0 |
| 70 | 6.0 | 10 | 23 | 7.9 | 35.9 ± 1.4 |
| 70 | 6.0 | 10 | 23 | 8.4 | 38.0 ± 1.5 |
| 70 | 6.0 | 10 | 37 | 7.4 | 30.6 ± 1.8 |
| 70 | 6.0 | 10 | 37 | 7.9 | 29.9 ± 0.7 |
| 70 | 6.0 | 10 | 37 | 8.4 | 29.5 ± 1.9 |

**Table S6** (continued). Diffusivity characterization data (mean ± SE).

| Dextran MW (kDa) | Stokes (Hydrodynamic) Radius (nm) | Concentration (mg/ml) | Polymerization Temperature (°C) | Polymerization pH | Diffusivity (µm^2^/s) |
| --- | --- | --- | --- | --- | --- |
| 150 | 8.5 | 4 | 23 | 7.4 | 50.2 ± 2.3 |
| 150 | 8.5 | 4 | 23 | 7.9 | 51.9 ± 2.5 |
| 150 | 8.5 | 4 | 23 | 8.4 | 51.5 ± 2.7 |
| 150 | 8.5 | 4 | 37 | 7.4 | 59.0 ± 8.6 |
| 150 | 8.5 | 4 | 37 | 7.9 | 43.9 ± 2.3 |
| 150 | 8.5 | 4 | 37 | 8.4 | 42.1 ± 0.9 |
| 150 | 8.5 | 6 | 23 | 7.4 | 52.6 ± 2.1 |
| 150 | 8.5 | 6 | 23 | 7.9 | 56.4 ± 3.2 |
| 150 | 8.5 | 6 | 23 | 8.4 | 54.4 ± 2.4 |
| 150 | 8.5 | 6 | 37 | 7.4 | 48.9 ± 1.6 |
| 150 | 8.5 | 6 | 37 | 7.9 | 42.1 ± 1.3 |
| 150 | 8.5 | 6 | 37 | 8.4 | 42.2 ± 2.6 |
| 150 | 8.5 | 8 | 23 | 7.4 | 43.9 ± 2.5 |
| 150 | 8.5 | 8 | 23 | 7.9 | 49.3 ± 1.8 |
| 150 | 8.5 | 8 | 23 | 8.4 | 41.0 ± 1.4 |
| 150 | 8.5 | 8 | 37 | 7.4 | 41.9 ± 1.7 |
| 150 | 8.5 | 8 | 37 | 7.9 | 32.3 ± 3.1 |
| 150 | 8.5 | 8 | 37 | 8.4 | 39.7 ± 2.2 |
| 150 | 8.5 | 10 | 23 | 7.4 | 40.5 ± 2.7 |
| 150 | 8.5 | 10 | 23 | 7.9 | 38.0 ± 1.5 |
| 150 | 8.5 | 10 | 23 | 8.4 | 41.6 ± 3.8 |
| 150 | 8.5 | 10 | 37 | 7.4 | 32.1 ± 3.4 |
| 150 | 8.5 | 10 | 37 | 7.9 | 34.4 ± 1.6 |
| 150 | 8.5 | 10 | 37 | 8.4 | 29.6 ± 1.6 |
