# Supplementary material for: Tunable Collagen I Hydrogels for Engineered Physiological Tissue Micro-Environments
Source: PLoS One. 2015 Mar 30;10(3):e0122500. doi: 10.1371/journal.pone.0122500 (PMC4378848; doi:10.1371/journal.pone.0122500)
Supplement: S5 Table — (DOCX) [file pone.0122500.s007.docx]

**S5 Table.** ANOVA results for polymerization half-time, compression modulus, pore diameter, and diffusivity.

| Property | Model | Parameter | Degrees of Freedom | Sum of Squares | F Ratio | p > F |
| --- | --- | --- | --- | --- | --- | --- |
| Polymerization Half-Time | **Full Factorial** | *C'* | 1 | 588.16 | 19.82 | **<.0001*** |
|  |  | *T'* | 1 | 1433.10 | 48.29 | **<.0001*** |
|  |  | *C'T'* | 1 | 373.96 | 12.60 | **0.0008*** |
|  |  | *pH'* | 1 | 2.44 | 0.08 | 0.7753 |
|  |  | *C'pH'* | 1 | 36.36 | 1.23 | 0.2731 |
|  |  | *T'pH'* | 1 | 3.82 | 0.13 | 0.7211 |
|  |  | *C'T'pH'* | 1 | 22.09 | 0.74 | 0.3919 |
|  |  | Model | 7 | 2293.60 | 11.04 |  |
|  |  | Error | 56 | 1661.96 |  |  |
|  |  | Total | 63 | 3955.56 |  |  |
|  | **Significant Effects** | *C'* | 1 | 588.16 | 20.52 | **<.0001*** |
|  |  | *T'* | 1 | 1433.10 | 50.00 | **<.0001*** |
|  |  | *C'T'* | 1 | 373.96 | 13.05 | **0.0006*** |
|  |  | Model | 3 | 2236.00 | 26.01 |  |
|  |  | Error | 60 | 1719.56 |  |  |
|  |  | Total | 63 | 3955.56 |  |  |

**S5 Table.** (continued)**.** ANOVA results for polymerization half-time, compression modulus, pore diameter, and diffusivity.

| Property | Model | Parameter | Degrees of Freedom | Sum of Squares | F Ratio | p > F |
| --- | --- | --- | --- | --- | --- | --- |
| Compression Modulus | **Full Factorial** | *C'* | 1 | 8.69E+08 | 118.46 | **<.0001*** |
|  |  | *T'* | 1 | 3.16E+08 | 43.13 | **<.0001*** |
|  |  | *C'T'* | 1 | 2.71E+07 | 3.70 | 0.0562 |
|  |  | *pH'* | 1 | 1.46E+08 | 19.85 | **<.0001*** |
|  |  | *C'pH'* | 1 | 4.02E+06 | 0.55 | 0.4602 |
|  |  | *T'pH'* | 1 | 3.17E+07 | 4.33 | 0.0390 |
|  |  | *C'T'pH'* | 1 | 7.56E+06 | 1.03 | 0.3114 |
|  |  | Model | 7 | 1.39E+09 | 27.16 |  |
|  |  | Error | 167 | 1.23E+09 |  |  |
|  |  | Total | 174 | 2.62E+09 |  |  |
|  | **Significant Effects** | *C'* | 1 | 8.52E+08 | 112.11 | **<.0001*** |
|  |  | *T'* | 1 | 3.20E+08 | 42.17 | **<.0001*** |
|  |  | *pH'* | 1 | 1.41E+08 | 18.59 | **<.0001*** |
|  |  | Model | 3 | 1.32E+09 | 57.97 |  |

**Table S7** (continued)**.** ANOVA results for polymerization half-time, compression modulus, pore diameter, and diffusivity.

| Property | Model | Parameter | Degrees of Freedom | Sum of Squares | F Ratio | p > F |
| --- | --- | --- | --- | --- | --- | --- |
| Pore Diameter | **Full Factorial** | *C'* | 1 | 5.44 | 11.25 | **0.0009*** |
|  |  | *T'* | 1 | 60.29 | 124.76 | **<.0001*** |
|  |  | *C'T'* | 1 | 0.14 | 0.30 | 0.5856 |
|  |  | *pH'* | 1 | 2.88 | 5.96 | **0.0153** |
|  |  | *C'pH'* | 1 | 0.63 | 1.31 | 0.2532 |
|  |  | *T'pH'* | 1 | 0.64 | 1.32 | 0.2509 |
|  |  | *C'T'pH'* | 1 | 2.43 | 5.03 | 0.0258 |
|  |  | Model | 7 | 72.45 | 21.42 |  |
|  |  | Error | 280 | 135.31 |  |  |
|  |  | Total | 287 | 207.77 |  |  |
|  | **Significant Effects** | *C'* | 1 | 5.44 | 11.10 | **0.0010*** |
|  |  | *T'* | 1 | 60.29 | 123.04 | **<.0001*** |
|  |  | *pH'* | 1 | 2.88 | 5.87 | **0.0160** |
|  |  | Model | 3 | 68.61 | 46.67 |  |
|  |  | Error | 284 | 139.16 |  |  |
|  |  | Total | 287 | 207.77 |  |  |

**Table S7** (continued)**.** ANOVA results for polymerization half-time, compression modulus, pore diameter, and diffusivity.

| Property | Model | Parameter | Degrees of Freedom | Sum of Squares | F Ratio | p > F |
| --- | --- | --- | --- | --- | --- | --- |
| Diffusivity | **Full Factorial** | *C'* | 1 | 3.55E+04 | 28.45 | **<.0001*** |
|  |  | *T'* | 1 | 1.25E+04 | 10.03 | **0.0016*** |
|  |  | *C'T'* | 1 | 1.21E+03 | 0.97 | 0.3248 |
|  |  | *pH'* | 1 | 2.51E+04 | 20.17 | **<.0001*** |
|  |  | *C'pH'* | 1 | 1.04E+03 | 0.84 | 0.3609 |
|  |  | *T'pH'* | 1 | 2.82E+02 | 0.23 | 0.6345 |
|  |  | *C'T'pH'* | 1 | 8.12E+03 | 6.51 | 0.0110 |
|  |  | *(R_H_^-1^)'* | 1 | 4.67E+06 | 3744.17 | **<.0001*** |
|  |  | *C'(R_H_^-1^)'* | 1 | 6.73E+03 | 5.40 | 0.0205 |
|  |  | *T'(R_H_^-1^)'* | 1 | 1.85E+03 | 1.48 | 0.2240 |
|  |  | *C'T'(R_H_^-1^)'* | 1 | 1.59E+03 | 1.28 | 0.2592 |
|  |  | *pH'(R_H_^-1^)'* | 1 | 7.50E+04 | 60.20 | **<.0001*** |
|  |  | *C'pH'(R_H_^-1^)'* | 1 | 5.55E+03 | 4.45 | 0.0353 |
|  |  | *T'pH'(R_H_^-1^)'* | 1 | 9.69E+02 | 0.78 | 0.3783 |
|  |  | *C'T'pH'(R_H_^-1^)'* | 1 | 1.30E+04 | 10.39 | 0.0130 |
|  |  | Model | 15 | 4.86E+06 | 259.75 |  |
|  |  | Error | 559 | 6.97E+05 |  |  |
|  |  | Total | 574 | 5.55E+06 |  |  |
|  | **Significant Effects** | *C'* | 1 | 3.55E+04 | 27.39 | **<.0001*** |
|  |  | *T'* | 1 | 1.25E+04 | 9.65 | **0.0020*** |
|  |  | *pH'* | 1 | 2.52E+04 | 19.44 | **<.0001*** |
|  |  | *(R_H_^-1^)'* | 1 | 4.67E+06 | 3601.94 | **<.0001*** |
|  |  | *pH'(R_H_^-1^)'* | 1 | 7.52E+04 | 58.04 | **<.0001*** |
|  |  | Model | 5 | 4.82E+06 | 743.03 |  |
|  |  | Error | 569 | 7.38E+05 |  |  |
|  |  | Total | 574 | 5.55E+06 |  |  |
